# Tooth wear development in the Australian Aboriginal dentition from Yuendumu: A longitudinal study

**DOI:** 10.1371/journal.pone.0254151

**Published:** 2021-07-09

**Authors:** Jinyoung Lee, Sarah Fung, Robin Yong, Sarbin Ranjitkar, John Kaidonis, Alistair R. Evans, Luca Fiorenza

**Affiliations:** 1 Department of Anatomy and Developmental Biology, Monash Biomedicine Discovery Institute, Monash University, Melbourne, Victoria, Australia; 2 Adelaide Dental School, University of Adelaide, Adelaide, South Australia, Australia; 3 School of Biological Sciences, Monash University, Melbourne, Victoria, Australia; 4 Geosciences, Museums Victoria, Melbourne, Victoria, Australia; 5 Earth Sciences, University of New England, Armidale, New South Wales, Australia; University of the Witwatersrand, SOUTH AFRICA

## Abstract

The analysis of dental wear, at both the microscopic and macroscopic scale, is one of the most widely used tools in archeology and anthropology to reconstruct the diet and lifestyle of past human populations. Biomechanical studies have indicated that tooth wear helps to dissipate the mechanical load over the crown surface, thus reducing the risk of tooth fracture. To date, there are only a few studies that have examined functional tooth wear variation in modern humans. Here we propose to study masticatory efficiency through the use of the Occlusal Fingerprint Analysis method, a well-developed digital approach that allows the reconstruction of the occlusal dynamics occurring during mastication. The aim of this study is to provide the first longitudinal quantitative data of molar and premolar macrowear patterns within a functional context. We examined the mixed and permanent dentition of one Australian Aboriginal child (from ages 8 to 17) from Yuendumu, using high-resolution surface scans of dental casts including both upper and lower arches. Our results suggest that the occlusal macrowear patterns of this individual did not significantly change through time. Occlusal contact parameters such as functional area, inclination and direction remain relatively unaltered throughout childhood and adolescence, indicating little change in the masticatory function of this individual. The functional tooth wear pattern in this individual did not change longitudinally indicating the degree of masticatory efficiency has most probably remained unaltered.

## Introduction

One of the primary roles of human teeth is to breakdown food into smaller pieces, providing an increased surface area for the digestive enzymes to act upon. The size and shape of teeth reflect the functional demands produced by the selective pressure of the physical properties of the ingested food [1]. Dental enamel is the hardest tissue in our body, and its main function is to protect the teeth from fracture and wear. However, tooth wear is an inevitable process. When the masticatory cycle starts, the contact of abrasive food particles between upper and lower tooth surfaces, and attritional wear between opposing teeth, produce the initial loss of tooth tissue and progression tooth wear. Although wear constantly changes and remodels the primary tooth morphology over the life of an individual, teeth still remain functional even with dentine exposure [2]. Thus, natural selection should also act on worn teeth, favouring teeth that wear in a way that keeps them mechanically efficient for processing foods [3]. Several studies have shown that teeth of primates and other mammals maintain efficiency for fracturing foods despite loss of dental tissue with age, thus preserving dental functionality throughout the wear sequence [4–8]. For example, the teeth of great apes maintain the ability to efficiently process plant foods throughout their long lifespans, even in more advanced wear stages [4, 7].

It is less clear, however, if the functional aspect of modern human teeth remains unchanged with wear. Studies that have evaluated if masticatory efficiency in modern humans changes with age have yielded wildly conflicting results. For example, some scholars suggest that masticatory performance, measured in relation to number of chewing strokes, surface areas, number of occlusal contacts and bite force, increases from the primary to the mixed dentition, and from the mixed to the permanent dentition [9–13]. This seems to be mostly associated with an increase in masticatory muscle thickness and to an enlargement of occlusal areas that are ultimately related to differences in body size. On the other hand, Ingervall [14, 15] has shown that the number of tooth contacts and the range of mandibular movements in children and adults are essentially the same, suggesting that adult levels in chewing efficiency are reached at 10 years of age. However, all these studies have been mostly based on the use of different age groups, and did not evaluate if masticatory efficiency changes within the same individuals over time. Moreover, while past studies have predominantly focussed on morphological features and functional aspects of occlusion, very little is known about how efficiency may vary as wear progresses [16].

In the present study, we propose to use a well-established method, called *Occlusal Fingerprint Analysis* (OFA) [17], to investigate if masticatory efficiency is maintained through time, despite the progressive loss of crown surface due to wear. Specifically, we examine dental functionality through a series of longitudinal dental samples belonging to the same individual: an Australian Aboriginal child from the Yuendumu collection [18]. Dental functionality has been measured in various ways using several dental topographic approaches, ranging from Geographic Information System (GIS) techniques [4] to Orientation Patch Count Rotated (OPCR) [19] and Dirichlet Normal Energy (DNE) [20]. Here, we apply the OFA method, a sophisticated digital approach that analyses and quantifies occlusal wear on tooth contacts, ranging from functional surfaces areas to spatial orientation [17]. The OFA method helps to reconstruct the occlusal mandibular movements responsible for the creation of wear contacts, and ultimately it provides essential information about masticatory behaviour of an individual [17]. It has been successfully employed to reconstruct the diet of historic and prehistoric human populations, and for the identification of unique cultural habits and oral pathologies [21–25].

By measuring parameters such as surface area, inclination and orientation of occlusal wear contacts of upper and lower teeth, we are able to detect any functional change occurred in the dentition over time. This information can help us to better understand how tooth wear advances, and how occlusal forces are distributed throughout the wear sequence, providing essential information about the variability of masticatory performance during dental development.

## Materials and methods

### Sample

For this project, we analysed the Yuendumu Aboriginal dental collection housed at the School of Dentistry at the University of Adelaide (Australia), selecting nine pairs of dental casts (taken annually from ages 8 to 17), including both upper and lower arches, belonging to the same individual (Δ549). This individual was selected because it was one of the best-preserved specimens within this collection, with an almost complete set of casts taken annually from 1960 until 1970. Moreover, this individual did not show any oral pathology and was characterized by clear wear contacts with well-defined margins, that are ideal for OFA.

This collection is one of the most widely studied dental samples in the world (with over 250 scientific publications), created from a unique longitudinal research project, where anthropologists annually examined the dentition and growth of Aboriginal children and young adults from Yuendumu in the Northern Territory between 1951 and 1971 [18]. The Yuendumu collection consists of measurements, radiographs, family data, and more importantly, 1717 sets of dental casts representing 446 individuals. The casts were made of dental stone, where a mixture of powder and water was allowed to set in dental impressions. This indigenous population was at an early stage of transition from a nomadic and hunter-gatherer way of life to a more settled existence, with limited contact with Europeans [18]. Their dentition was mostly characterised by a normal occlusion (or Angle Class I), with little evidence of pathological conditions, such as caries, tooth crowding, malocclusions, molar agenesis and periodontal diseases. The rate of tooth wear was extensive, and it was mostly due to the consumption of a coarse diet and to the vigorous use of the dentition for non-masticatory purposes (i.e. tool use) [26, 27]. As a result, their wear patterns were dominated by clear and large wear areas characterised by sharp and steep edges [28]. We selected permanent molars and premolars, and deciduous molars with a degree of wear comprised between 1 (no dentine exposure and small wear areas) and 4 (full cusp removal with several large dentine exposures), according to the scoring system created by Smith [29] (S1 Table in S1 File). The OFA method requires each individual tooth to be assessed separately due to its unique pattern (hence the name fingerprint) [17]. The sample includes eight deciduous molars, eight permanent premolars and 12 permanent molars: 28 teeth for nine years for the individual tooth classes, giving a total number of 130 teeth examined individually (S1 Table in S1 File). The use of Yuendumu dental casts for this study is covered by the approval from the Human Ethics Research Committee, University of Adelaide (H-27-1990).

### Data acquisition

**Fig 1:**
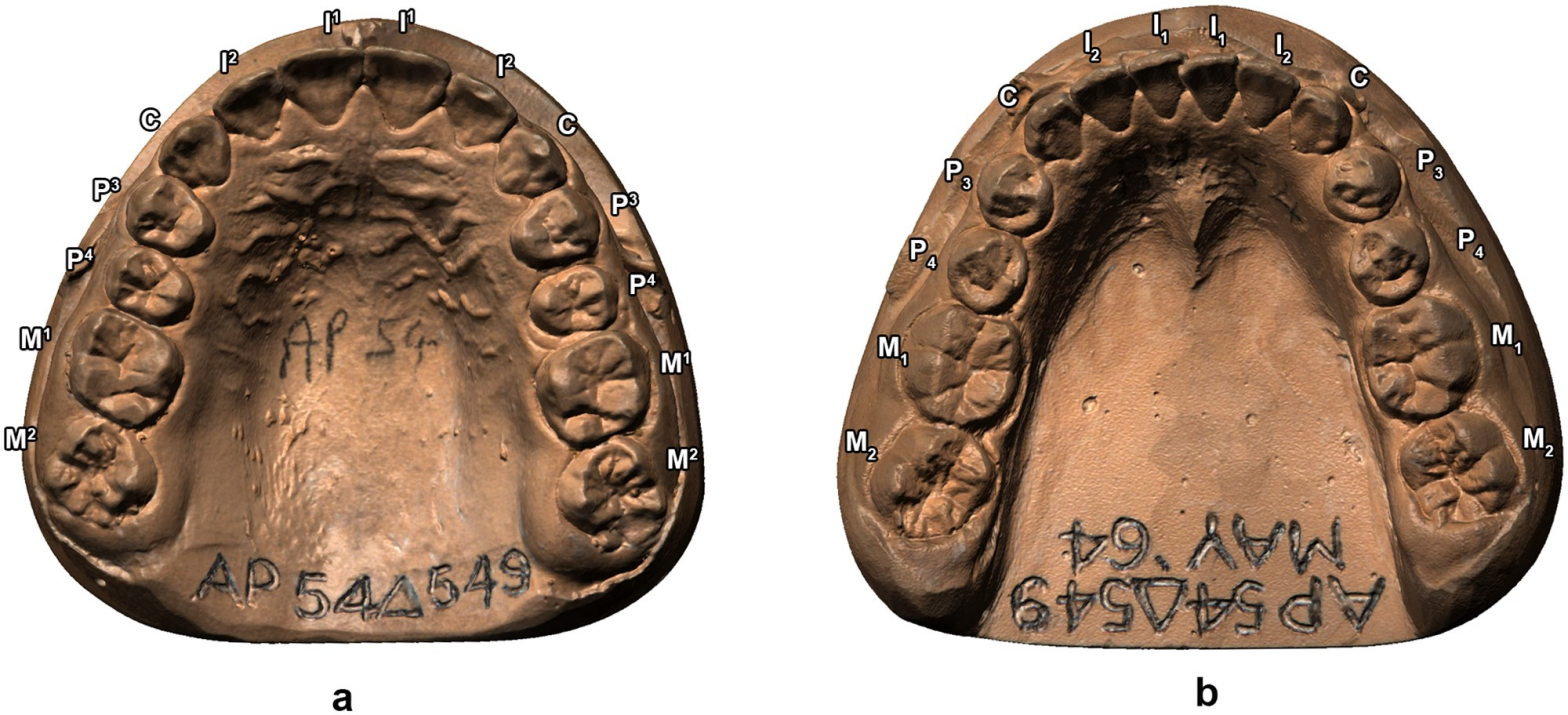
Three-dimensional digital models of upper (a) and lower (b) dental arches of a 12-year old Aboriginal child (Δ549). Images not to scale.

Casts of upper and lower dental arches were digitised using a white-light scanning system based on structured-light technology with a *xy* resolution of 45 μm (smartSCAN3D C-5, Breuckmann, GmbH) (Fig 1).

Collection and alignment of the scan-data was carried out using the integrated scanning software optoCAT (Breuckmann, GmbH). Different views acquired with the surface scanner were manually aligned by selecting three homologous points on each image and then instructing the system to compute a best fit alignment using a maximum point distance of 0.5 mm, until a sub-sampling ratio of 1/1 was reached [25]. The 3D virtual models were further post-processed using PolyWorks^®^ V12 (InnovMetric Software Inc.), a 3D metrology platform software. The unfinished polygonal model was imported into the IMEdit module where topology errors, artifacts, and degenerate/duplicate triangles were manually identified and removed.

### 3D printing

In addition, we generated high-resolution physical replicas of the digital models via stereolithography, since 3D prints can be more helpful when trying to identify qualitative details, such as cusp morphology, fissure patterns and wear areas than a two-dimensional photograph or a 3D model viewed on a screen [30]. Furthermore, we tested the presence of tooth-to-tooth occlusal contact in the intercuspal position between upper and lower teeth using an 8 μm Shimstock Foil (Coltene^™^) [31] that was inserted between 3D prints of upper and lower dental arches [32].

### Occlusal Fingerprint Analysis (OFA)

**Fig 2:**
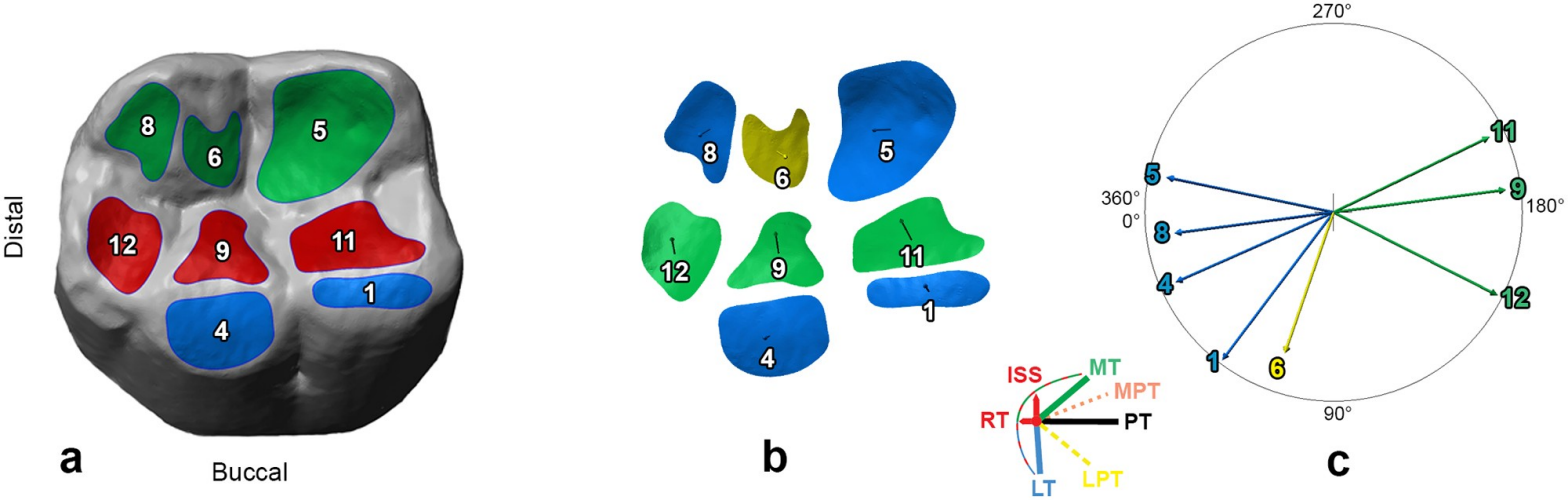
Occlusal fingerprint analysis: Contact areas identification (*a*), directional vectors (*b*), and three-dimensional occlusal compass (*c*). In (*a*) macrowear areas of a first lower permanent molar grouped by chewing cycle phases [31]: Buccal phase I areas (in blue; 1, 1.1, 2, 2.1, 3 and 4), Lingual phase I areas (in green; 5, 5.1, 6, 6.1, 7 and 8), and Phase II areas (in red; 9, 10, 11, 12 and 13). In (*b*) and (*c*) wear areas are colour-coded based on the *Dental Occlusal Concept* [35], which identifies four major occlusal movements: lateroretrusive (LRT) areas (in blue; 1, 1.1, 5, 5, 5.1 and 8), lateroprotrusive (LP) areas (in yellow; 2, 2.1, 3, 6, 6.1 and 7), mediotrusive and immediate side shift (MT + ISS) areas (in green; 9, 11 and 12), and medioprotrusive (MPT) areas (in orange; 10 and 13).

OFA is a digital approach used to describe and quantify occlusal macrowear patterns through the analysis of surface area, inclination and spatial orientation of individual wear contact areas [17]. This method is based on five sequential steps (Fig 2):

1. **Model orientation**. The polygonal models of upper and lower dental arches are aligned and oriented using the occlusal plane (defined by three landmarks selected on the lowest surface point of the most posterior molar and central incisor), which is later translated into the *xy* coordinate system [22].
2. **Contact areas identification**. Wear contact areas are manually identified onto the polygonal model following the labelling system created by Maier and Schneck [33] and later modified by Kullmer and colleagues [17], who recognised 13 pairs of homologous wear areas in modern human molars (Fig 2a).
3. **Surface area**. As larger teeth likely develop larger contact areas than smaller teeth, we use relative areas to minimise variation in size between the different tooth classes. Relative wear contact areas (in %) are obtained by dividing the surface area of Buccal, Lingual and Phase II areas with the total occlusal wear. Surface areas are automatically calculated in mm^2^ by selecting all the triangles enclosed within a specific wear contact using the *area* function available in Polyworks^®^ V12, (InnovMetric Software Inc.).
4. **Inclination**. The inclination is simply the angle measured between the contact’s plane and the reference (occlusal) plane. The contact plane is created by selecting all digital surface points delimited within the contact area’s perimeter, and by applying the *best-fit plane* function of Polyworks^®^ V12, (InnovMetric Software Inc.).
5. **Spatial orientation**. The directional data is obtained by creating vectors that are perpendicular to the contact’s plane (Fig 2b), grouping them according to the *dental occlusal concept* [34]. The vectors are subsequently projected onto the reference (occlusal) plane. The three-dimensional occlusal compass is created by translating the vectors (which are enclosed within a circle) to an arbitrary point (*x* = 0, *y* = 0, *z* = 0) with a standardised length (Fig 2c).

### Statistical analysis

We employed exploratory statistical analyses, such as median, standard deviation and interquartile ranges, to describe the proportions of Buccal, Lingual (phase I) and Phase II areas, and the wear plane angles. The relative surface areas of wear contacts were visually described through ternary diagrams, which are triangular coordinate systems that illustrate the ratios of three variables that sum to 1 or 100% [35]. For the between-group comparisons we used the one-way PERMANOVA test, a non-parametric test of significant differences between two or more groups, which is calculated directly from any asymmetric distance or dissimilarity matrix [36]. Statistical significance was computed with a permutation test of group membership (n = 9999).

Because most statistical methods are not directly applicable to directional data [35], we have employed circular statistical tools for the analysis of wear contact directions [37], considering the circular mean angle, the circular standard deviation and the 95% confidence interval. Furthermore, we tested if our directional data indicated any deviation of the circular distribution from a perfect circle by measuring the concentration parameter (κ), which explains how concentrated the distribution around the mean is, and by executing the Rayleigh’s and Rao’s Spacing tests for uniformity [38–40]. The linear statistical analysis and ternary plots were acquired with the software PAST v.3.22 (PAlaeontological Statistics) [41], while directional statistic results were obtained using a circular statistic software program (Oriana^™^ v. 4.00, Kovach Computing Services).

## Results

The macrowear pattern of the individual analysed in this study is mostly dominated by Phase II areas (40%), followed by Buccal phase I areas (35%), and by Lingual phase I areas (25%) (Table 1).

**Table 1:** Descriptive statistics of relative wear areas of posterior teeth, including age, sample number (N), median, standard deviation (SD) and interquartile range (IQR).

Sample	N	Buccal phase I			Lingual phase I			Phase II		
		Median	SD	IQR	Median	SD	IQR	Median	SD	IQR
Age 8	12	0.33	0.11	0.15	0.32	0.11	0.17	0.36	0.08	0.13
Age 9	10	0.36	0.14	0.21	0.32	0.09	0.15	0.36	0.10	0.19
Age 11	12	0.34	0.11	0.21	0.23	0.14	0.22	0.43	0.16	0.29
Age 12	16	0.35	0.12	0.13	0.20	0.15	0.24	0.45	0.12	0.14
Age 13	16	0.35	0.07	0.07	0.24	0.12	0.17	0.41	0.10	0.14
Age 14	16	0.34	0.09	0.16	0.25	0.12	0.12	0.41	0.11	0.16
Age 15	16	0.35	0.10	0.14	0.25	0.13	0.16	0.39	0.09	0.12
Age 16	16	0.37	0.15	0.18	0.23	0.14	0.22	0.40	0.11	0.22
Age 17	16	0.35	0.14	0.14	0.23	0.15	0.30	0.42	0.11	0.11

**Fig 3:**
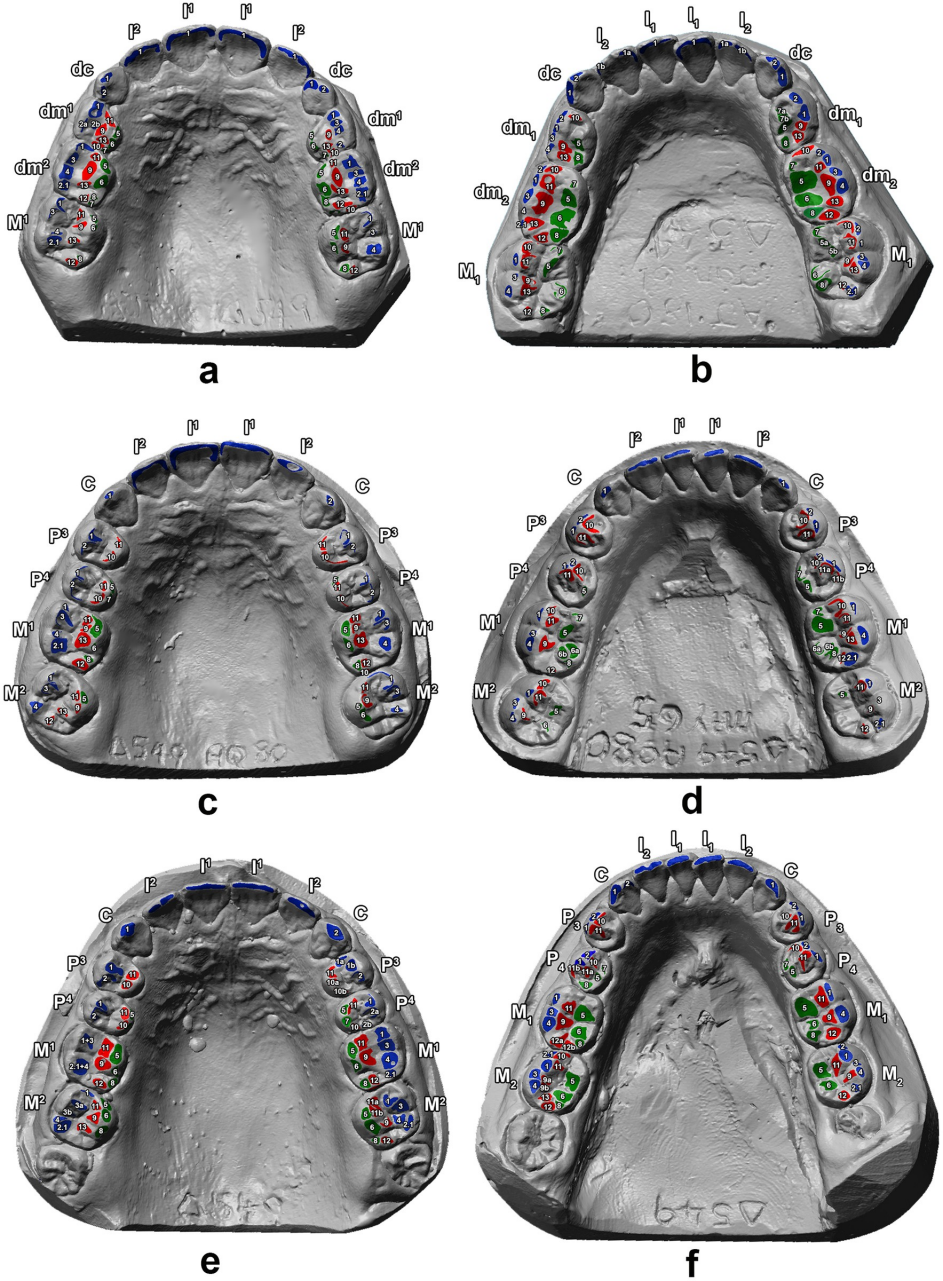
Three-dimensional digital models of upper and lower arches showing the macrowear patterns. Wear contacts have been labelled following the numbering system created by Maier & Schneck [30] and grouped by chewing cycle phases [31, 32]: Buccal phase I (areas 1, 1.1, 2, 2.1, 3 and 4; coloured in blue), Lingual phase I (areas 5, 5.1, 6, 6.1, 7 and 8; coloured in green) and Phase II (areas 10, 11, 12 and 13; coloured in red). CA1 upper (*a*) and lower (*b*) arches, age E 8.07 years; CA4 upper (*c*) and lower (*d*) arches, age E 12.44 years; CA9 upper (*e*) and lower (*f*) arches, age E 17.44 years.

Phase II areas show the least degree of variation, while Lingual phase I areas vary the most, especially in the later ages (16 and 17). There is a general increase in absolute areas from the mixed dentition to the adult dentition (Fig 3). However, the proportion of shearing, crushing and grinding wear does not seem to vary during childhood and adolescence. This is further confirmed by the multivariate statistical analysis (one-way PERMANOVA, Table 2) showing no significant differences between the various age groups, with the exception of the pairwise comparison between age 8 and age 12 (*p* = 0.02).

**Table 2:** Multivariate statistical analysis of differences in relative wear contact areas of the different sets grouped according to chewing cycle phases [31].

**Set**	**Age 8**	**Age 9**	**Age 11**	**Age 12**	**Age 13**	**Age 14**	**Age 15**	**Age 16**	**Age 17**
Age 8	-								
Age 9	0.68	-							
Age 11	0.15	0.24	-						
Age 12	**0.02**	0.06	0.81	-					
Age 13	0.10	0.20	0.91	0.53	-				
Age 14	0.12	0.25	0.88	0.53	0.99	-			
Age 15	0.24	0.44	0.72	0.31	0.82	0.90	-		
Age 16	0.11	0.34	0.68	0.52	0.74	0.79	0.75	-	
Age 17	0.12	0.24	0.97	0.79	0.96	0.94	0.76	0.80	-

Note. One-way permutational multivariate analysis of variance test,

Permutation N = 9,999, P (same) = 0.5996.

Significantly *p* values (< 0.05) are highlighted in bold.

**Fig 4:**
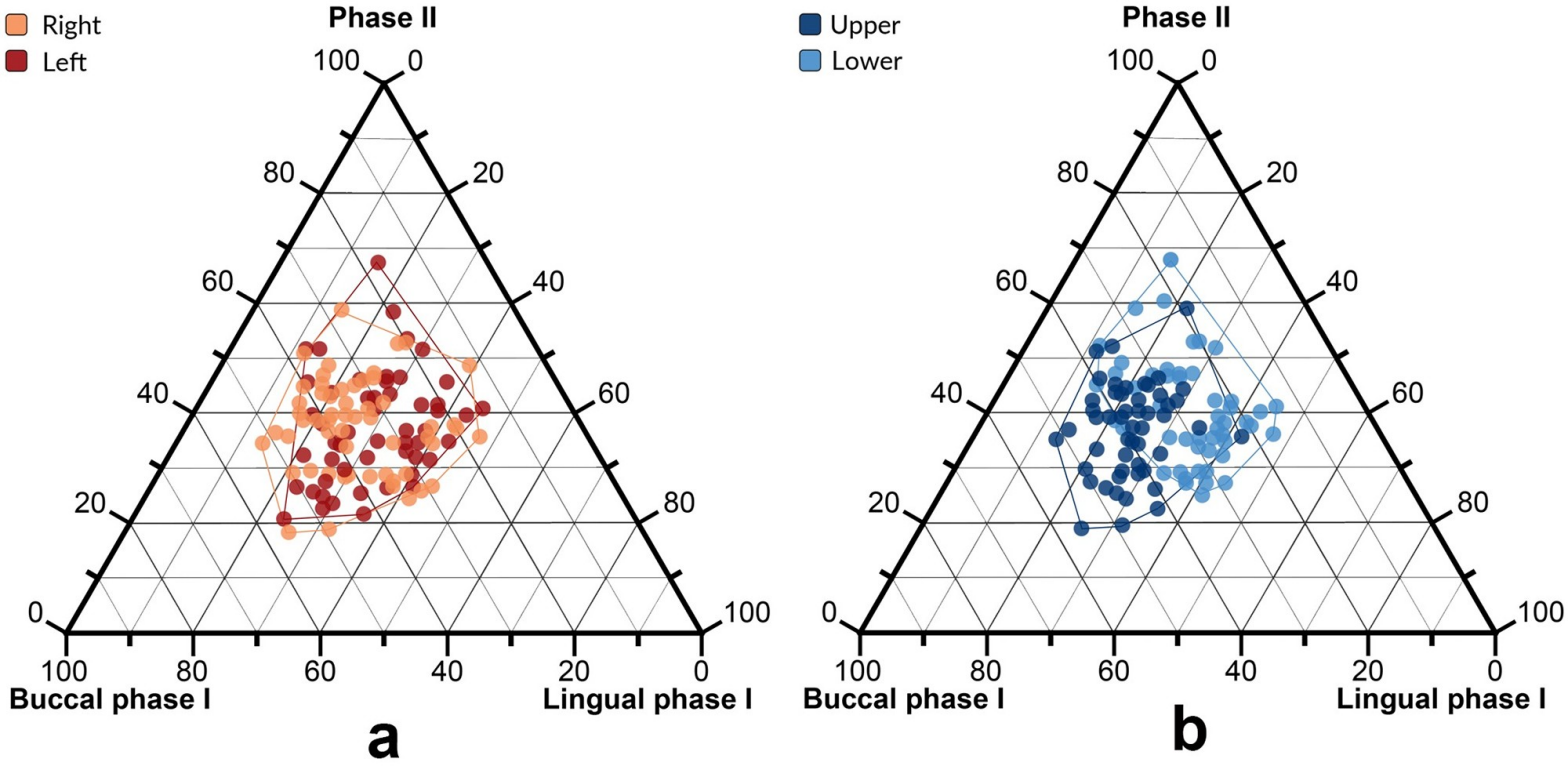
Ternary diagrams showing the proportions (in %) of relative wear areas comparing left and right (*a*), and upper and lower teeth (*b*). The three variables (Buccal phase I, Lingual phase I and Phase II areas) are positioned in an equilateral triangle. Each base of the triangle represents a ratio of 0% while the vertices correspond to a percentage of 100%.

We also investigated the possible presence of masticatory asymmetry [42], by comparing the macrowear patterns of the right posterior teeth with those of the left side, and by comparing the occlusal contact areas between antagonist teeth (Fig 4). We did not find any statistically significant difference between left and right teeth for all the ages considered in this study (one-way PERMANOVA, *p* > 0.05). Some statistically significant differences were found between upper and lower arches at age 8 (*p* = 0.003), age 9 (*p* = 0.008) and age 17 (*p* = 0.040). These differences are mostly driven by Buccal phase I areas (S2 Table in S1 File), while Phase II areas of upper teeth do not statistically differ from the wear areas of lower teeth.

We have also considered wear variation through time by mapping the absolute surface area of each occlusal contact at each age (S1–S4 Figs in S1 File). These graphs show a general increase in wear surface areas in older ages. We observed a more prominent increase in Phase II contacts compared to Phase I wear areas.

For the analysis of the inclination we divided the sample into different wear stage groups (1, 2 and 3) [29], since previous studies have shown that wear plane angles significantly decrease in more advanced wear stages [21]. We noticed that Phase II areas are generally characterised by flatter angles, especially in more advanced wear stages (Table 3). Buccal phase I contact planes are steeper than Lingual phase I areas in wear stages 1 and 3, while the opposite situation is found in wear stage 2. No statistical differences have been found between ages in wear stage 1, except for ages 8 and 9 (one-way PERMANOVA, *p* = 0.0172). In wear stage 2, there was no statistically significant difference between any ages, while in wear stage 3, we found statistically significant difference only between ages 8 and 16 (*p* = 0.0488).

**Table 3:** Descriptive statistical analysis of wear contact inclinations of posterior teeth grouped according to their wear stage [34], including sample number (N), median, standard deviation (SD) and interquartile range (IQR).

WEAR STAGE 1		Buccal phase I			Lingual phase I			Phase II		
Set	N	Median	SD	IQR	Median	SD	IQR	Median	SD	IQR
Age 8	4	31.07	2.93	5.41	30.07	2.77	4.59	35.05	1.73	3.28
Age 9	5	26.23	2.73	4.66	22.77	5.53	10.10	27.41	8.74	10.88
Age 11	10	27.92	9.30	16.36	29.84	6.77	11.84	31.86	6.90	10.41
Age 12	8	26.14	6.90	7.96	30.25	6.77	20.27	30.85	9.30	14.16
Age 13	4	37.90	13.92	26.52	25.8	5.5	10.2	34.77	3.35	5.28
Age 14	4	42.19	17.82	34.13	28.14	4.60	8.47	34.40	5.87	10.84
Age 15	4	42.26	17.62	32.47	29.13	6.22	11.63	31.07	5.32	9.94
Age 16	4	35.43	9.48	18.28	28.43	1.88	3.61	28.98	6.45	11.59
Age 17	-	-	-	-	-	-	-	-	-	-
**WEAR STAGE 2**										
Age 8	2	33.36	1.34	1.42	39.65	4.00	4.24	11.82	0.81	0.86
Age 9	-	-	-	-	-	-	-	-	-	-
Age 11	-	-	-	-	-	-	-	-	-	-
Age 12	3	27.99	5.23	9.44	26.98	7.81	14.60	27.68	2.60	4.73
Age 13	9	31.35	8.09	9.16	30.25	17.61	31.74	25.77	5.92	8.93
Age 14	9	30.22	8.09	10.65	33.83	17.61	20.31	28.13	5.92	13.05
Age 15	6	33.48	13.69	18.01	37.99	12.83	21.57	32.03	7.04	13.65
Age 16	6	33.42	24.44	48.55	35.16	20.92	40.63	22.66	13.47	26.90
Age 17	4	26.57	5.82	10.76	29.38	5.09	9.70	34.26	2.65	4.43
**WEAR STAGE 3**										
Age 8	6	29.87	5.45	8.36	32.67	7.80	12.55	16.84	1.85	3.44
Age 9	4	29.20	8.19	14.94	31.98	7.34	12.62	16.24	4.39	8.32
Age 11	-	-	-	-	-	-	-	-	-	-
Age 12	-	-	-	-	-	-	-	-	-	-
Age 13	-	-	-	-	-	-	-	-	-	-
Age 14	2	36.44	19.37	20.54	27.44	4.89	5.18	18.94	4.80	5.09
Age 15	4	32.16	10.88	18.76	27.62	6.93	12.33	21.06	5.17	9.63
Age 16	4	25.44	11.36	19.20	27.07	11.35	19.05	18.14	7.66	13.02
Age 17	6	32.26	6.51	6.41	26.32	13.31	14.38	20.15	2.95	3.42

Finally, we examined whether the vectors described by the *dental occlusal concept* [34] follow a normal distribution or point toward a preferred direction (Table 4). The circular statistical analysis shows that all the major occlusal movements considered in this study, for any given age, fall within a von Mises distribution (the equivalent of a normal distribution for linear data), displaying preferred directions with significant values for both the Rayleigh’s test and Rao’s spacing test (*p* < 0.05). We did not find any statistically significant difference (Watson-Williams F-test) when we compared the directional angles between the different ages (S3 Table in S1 File).

**Table 4:** Descriptive circular statistical analysis of wear contact directions, including the circular mean, the circular standard deviation (SD), the concentration parameter (κ), and the Rayleigh test.

**Age**	**LRT**					**LPT**				
	**N**	**Mean**	**SD**	**K**	**Rayleigh** (P)	**N**	**Mean**	**SD**	**K**	**Rayleigh** (P)
Age 8	6	71.21	6.07	50.49	**2E-05**	6	136.13	24.18	3.47	0.085
Age 9	5	83.63	9.86	16.86	**0.000**	5	141.99	25.64	2.73	**0.008**
Age 11	5	89.90	64.96	0.91	0.263	5	149.37	37.17	1.47	**0.028**
Age 12	7	84.88	33.33	2.18	**0.003**	7	148.98	49.70	1.78	**0.030**
Age 13	8	57.33	34.21	2.24	**0.001**	8	149.63	51.94	1.66	**0.024**
Age 14	8	81.26	39.13	1.83	**0.003**	8	151.52	28.79	2.98	**4E-04**
Age 15	8	90.09	43.42	1.56	**0.007**	8	140.71	27.09	3.31	**2E-04**
Age 16	8	96.02	38.70	1.86	**0.003**	8	134.26	42.80	1.59	**0.006**
Age 17	8	100.14	44.56	1.50	**0.008**	8	140.43	32.02	2.49	**8E-04**
**Age**	**MT/ISS**					**MPT**				
	**N**	**Mean**	**SD**	**K**	**Rayleigh** (P)	**N**	**Mean**	**SD**	**K**	**Rayleigh** (P)
Age 8	6	294.29	24.78	1.41	**0.002**	6	267.51	56.92	3.23	0.104
Age 9	5	299.71	52.55	1.38	0.114	5	229.15	21.86	48.29	**0.005**
Age 11	5	303.39	51.27	28.79	0.103	5	251.66	10.46	1.74	**0.002**
Age 12	7	289.56	63.15	4.87	0.125	5	260.18	25.82	0.89	**0.008**
Age 13	8	252.03	70.26	17.43	0.171	8	252.78	29.85	0.97	**5E-04**
Age 14	8	277.30	73.95	5.59	0.226	6	247.64	29.15	0.71	**0.004**
Age 15	8	280.87	71.72	3.01	0.192	6	240.40	34.80	0.74	**0.009**
Age 16	8	275.86	68.16	5.15	0.144	8	266.50	36.53	0.74	**0.002**
Age 17	8	271.12	57.99	1.68	0.052	7	258.03	51.39	0.74	**0.037**

* Directions: LRT (lateroretrusion), LPT (lateroprotrusion), MT/ISS (mediotrusion and immediate sideshift) and MPT (medioprotrusion).

Significant values (*p* < 0.05) are highlighted in bold.

## Discussion

Our results show that the occlusal macrowear patterns of this individual did not significantly change through time. Occlusal contact parameters such as functional area, inclination and directional angles remain relatively unaltered throughout childhood and adolescence, indicating little change in the masticatory function during this period of time.

A macrowear pattern dominated by Phase II areas, followed by Buccal phase I areas, is generally consistent with a highly abrasive diet composed of meat and plant foods that both require grinding and shearing [43, 44]. Australian Aborigines at Yuendumu Settlement were at an early stage of transition from a nomadic hunter-gathering lifestyle [18]. They consumed a wide range of foods, including western foods, that contained flour and sugar, but also coarse and fibrous native foods that required vigorous and prolonged mastication [18, 26, 27, 45]. Their extensive occlusal wear was also caused by grit and dust present in their desert environment and by the way the food was prepared [18, 46]. For example, many foods were eaten raw or received minimal cooking either on an open fire or through an earth oven of hot sand and ashes [47]. Teeth were also used as a vice or a third hand for cutting, holding, and shaping objects [18]. These cultural habits favoured the occasional ingestion of exogenous and abrasive material, which in turn may have significantly increased the amount of tooth tissue loss [18, 25, 47].

The absence of significant macrowear differences between the different ages of this individual suggests the presence of vigorous mastication since childhood. This is consistent with earlier observations that described extensive occlusal wear in the deciduous dentition of Aboriginal children before first permanent molars erupted [27, 48]. Although the Aboriginal children from Yuendumu were breastfed until relative late (between 3 and 5 years), they were given hard foods that required prolonged chewing from an early age [26]. The increase in absolute contact wear areas through time is expected and consistent with previous observations [49]. We observe a general increase in Phase II areas in more advanced wear stages to the detriment of Buccal wear areas. Similar patterns have been observed in hunter-gatherer populations with a highly abrasive diet [50].

The dentition of the Aboriginal people from Yuendumu was characterised by symmetric wear, with one side being almost a mirror image of the other [26]. Our quantitative analysis confirms these earlier annotations, showing that macrowear patterns of the left side of this individual are very similar to those of the right counterpart at any age considered in this study. Direct observations and analysis of cinematographic records revealed that Yuendumu people chewed on one side at a time, alternating between right and left sides with striking regularity [18, 26]. This unilateral interdigitation, typical of the Yuendumu population, was described as *X-occlusion*, or *alternate intercuspation*, and it allowed a much wider range of lateral masticatory movements, which are particularly advantageous when grinding and chewing tough foods [18, 26, 51]. It has been suggested that a divergent pattern of growth of the maxillary and mandibular dental arches would lead to alternate intercuspation [51]. Differences in morphology of upper and lower teeth, together with disparity between upper and lower dental arch breadths in Yuendumu people probably explains the difference we found in macrowear patterns of this individual between upper and lower teeth, especially in the youngest ages. Similar results were found in another study that investigated molar inclination in the Aboriginal people from Yuendumu [42]. The wide masticatory movements of this individual remained relatively unaltered throughout childhood and adolescence, confirming the results of a previous study that examined the range of movement of the mandible in Swedish children, showing that the distribution of protrusion and lateral movement of 10-year-old children was similar to those of adults [14].

Although other studies have shown that bite force capacity, occlusal surface area, and number of occlusal contacts increase with age [9–13], they do not necessarily relate to an improvement in masticatory efficiency. For example, the number of occlusal contacts does not necessarily provide information about their functional aspects. With the OFA approach we are able to reconstruct the occlusal movements responsible for the formation of these wear areas, and therefore we can provide a more accurate picture of the relationship between masticatory patterns and occlusal wear. Possessing large occlusal surface areas decreases the efficiency when processing foods with higher toughness and Young’s modulus, as the greater areas spread out the overall bite force applied to food particles [52]. Maximum bite force undoubtedly increases throughout childhood and adolescence. However, the increase in bite force is proportionally correlated to an increase in body size [53], and alone it may not be a valid indicator of masticatory performance. Interestingly, a study that measured the dimensions of comminuted food particles in children of different ages did not find any significant correlation between masticatory efficiency and body weight [16]. However, we should be careful in comparing masticatory efficiency across populations with different diets and different lifestyles. The amount of tooth wear, occlusal relationships and chewing behaviour differ significantly between indigenous and industrialized populations [54].

Overall, our study shows that dental functionality and masticatory patterns in this individual remains relatively unaltered despite wear. The change from a mixed to a permanent dentition does not seem to have had any significant impact on dental functionality, further confirming earlier observations on masticatory patterns in the Yuendumu people [18, 26, 47, 51, 55].

Biomechanical studies have shown that tooth wear seems to minimise tensile stresses in the tooth crown, thus preventing potential fractures in the enamel layer [56–58]. Ultimately, this helps in maintaining masticatory efficiency throughout the lifetime of an individual. For example, it has been suggested that the smoothing of the occlusal surface from the consumption of tough and pliant food sources in orangutans diminishes the local contact stresses and re-distributes the tensile stresses to the cervical margins, which inhibits deep cracks but favours margin fractures [59–61].

Moreover, the lack of dental wear in industrialised societies might be a primary factor leading to non-carious cervical lesions [56]. This would indicate that tooth wear has probably played an important role in the evolution of mammalian dentitions, enabling teeth to be mechanically efficient throughout the lifetime of an individual [57]. Another interesting aspect that has not yet been investigated it is how malocclusions affect the occlusal dynamics and masticatory movements. Previous studies have shown that in altered occlusions there is a decrease in occlusal contact areas that negatively influences masticatory efficiency and maximum bite forces [61]. However, dental wear patterns have been little studied in individuals characterized by different occlusal variations. We have analyzed the dentition of an adult male gorilla characterized by the presence of a supernumerary upper premolar, showing that the occlusal contacts could not be associated with a normal chewing behaviour [62]. This gorilla was probably affected by malocclusions that may have caused discomfort during chewing, probably leading to attritional tooth grinding.

To further confirm our interpretation of the results of this study, it is necessary to expand the sample size including individuals with older ages. In addition, it will need to be tested whether the OFA method can correlate with measures of masticatory function in living individuals. A previous study analysed the dentition of a 48-year-old female with detailed information about nutrition and daily eating behaviour, showing a good correlation between OFA parameters and masticatory function [17]. However, for a better understanding on how masticatory function changes through time it will be necessary to carry out OFA longitudinal experiments using modern humans with different dietary habits.

Although our current study was limited to the analysis of one single individual, and did not include information about advanced wear stages, it is the first such longitudinal study of tooth wear to be carried out. Some studies have suggested that dental senescence in certain primate species compromises the ability to chew effectively through foods, and ultimately affecting their reproductive success [63]. As such, future studies could investigate if masticatory function in modern humans is maintained throughout the wear sequence by employing a larger sample size, ideally from populations with different dietary habits. Furthermore, biomechanical analyses could provide additional information about the effect of age and maturation on how dental traits respond to stress and strains produced during different masticatory loads.

## Supporting information

S1 File (DOCX)

S1 Data (XLSX)
