# Supplementary material for: Tooth wear development in the Australian Aboriginal dentition from Yuendumu: A longitudinal study
Source: PLoS One. 2021 Jul 9;16(7):e0254151. doi: 10.1371/journal.pone.0254151 (PMC8270463; doi:10.1371/journal.pone.0254151)
Supplement: S1 File — (DOCX) [file pone.0254151.s001.docx]

**S1 File**

**Title: Functional tooth wear in the Australian Aboriginal dentition from Yuendumu: A longitudinal study**

Jinyoung Lee^1^, Sarah Fung^1^, Robin Yong^2^, Sarbin Ranjitkar^2^, John Kaidonis^2^, Alistair R. Evans^3,4^, Luca Fiorenza^1,5^

^1^ *Monash Biomedicine Discovery Institute,* *Department of Anatomy and Developmental Biology, Monash University, Melbourne, Victoria, Australia*

^2^ *Adelaide Dental School, University of Adelaide, Adelaide, South Australia, Australia*

^3^ *School of Biological Sciences, Monash University, Melbourne, Victoria, Australia*

^4^ *Geosciences, Museums Victoria, Melbourne, Victoria, Australia*

^5^ *Earth Sciences, University of New England, Armidale, New South Wales, Australia*

**S1 Table.** List of dental sets (from the specimen Δ549) analysed in this study, including information about of age (E = estimated), number of teeth examined (N) and tooth type.

| **Age** | **N** | **Arch** | **Teeth** |
| --- | --- | --- | --- |
| E 8.07 | 12 | Upper | Ldm^1^, Rdm^1^, Ldm^2^, Rdm^2^, LM^1^, RM^1^ |
|  |  | Lower | Ldm_1_, Rdm_1_, Ldm_2_, Rdm_2_, LM_1_, RM_1_ |
| E 9.65 | 10 | Upper | Ldm^1^, Ldm^2^, Rdm^2^, LM^1^, RM^1^ |
|  |  | Lower | Ldm_1_, Ldm_2_, Rdm_2_, LM_1_, RM_1_ |
| E 11.44 | 12 | Upper | LP^3^, RP^3^, RP^4^, LM^1^, RM^1^, LM^2^ |
|  |  | Lower | LP_3_, RP_3_, RP_4_, LM_1_, RM_1_, LM_2_ |
| E 12.44 | 16 | Upper | LP^3^, RP^3^, RP^4^, LP^4^, LM^1^, RM^1^, LM^2^, RM^2^ |
|  |  | Lower | LP_3_, RP_3_, LP_4_, RP_4_, LM_1_, RM_1_, LM_2_, RM_2_ |
| E 13.44 | 16 | Upper | LP^3^, RP^3^, RP^4^, LP^4^, LM^1^, RM^1^, LM^2^, RM^2^ |
|  |  | Lower | LP_3_, RP_3_, LP_4_, RP_4_, LM_1_, RM_1_, LM_2_, RM_2_ |
| E 14.44 | 16 | Upper | LP^3^, RP^3^, RP^4^, LP^4^, LM^1^, RM^1^, LM^2^, RM^2^ |
|  |  | Lower | LP_3_, RP_3_, LP_4_, RP_4_, LM_1_, RM_1_, LM_2_, RM_2_ |
| E 15.44 | 16 | Upper | LP^3^, RP^3^, RP^4^, LP^4^, LM^1^, RM^1^, LM^2^, RM^2^ |
|  |  | Lower | LP_3_, RP_3_, LP_4_, RP_4_, LM_1_, RM_1_, LM_2_, RM_2_ |
| E 16.44 | 16 | Upper | LP^3^, RP^3^, RP^4^, LP^4^, LM^1^, RM^1^, LM^2^, RM^2^ |
|  |  | Lower | LP_3_, RP_3_, LP_4_, RP_4_, LM_1_, RM_1_, LM_2_, RM_2_ |
| E 17.44 | 16 | Upper | LP^3^, RP^3^, RP^4^, LP^4^, LM^1^, RM^1^, LM^2^, RM^2^ |
|  |  | Lower | LP_3_, RP_3_, LP_4_, RP_4_, LM_1_, RM_1_, LM_2_, RM_2_ |

**S2 Table.** Between-group comparisons of relative wear areas of upper and lower teeth.

| **Age** | **Buccal phase I** | **Lingual phase I** | **Phase II** |
| --- | --- | --- | --- |
| Age 8 | **0.002** | **0.002** | 0.940 |
| Age 9 | **0.008** | **0.016** | 0.222 |
| Age 11 | **0.026** | 0.981 | 0.221 |
| Age 12 | **0.035** | 0.692 | 0.267 |
| Age 13 | 0.077 | 0.368 | 0.940 |
| Age 14 | **0.010** | 0.289 | 0.243 |
| Age 15 | **0.027** | 0.203 | 0.458 |
| Age 16 | **0.015** | 0.097 | 0.742 |
| Age 17 | **0.007** | 0.148 | 0.224 |

Note. Mann-Whitney U test,

P values adjusted with Bonferroni Correction

Significantly p values (< 0.05) are highlighted in bold.

**S3 Table.** Pairwise comparisons of wear contact directions grouped according to the major occlusal movements (Douglas and De Vreugd, 1997).

| **LRT** | **Age 8** | **Age 9** | **Age 11** | **Age 12** | **Age 13** | **Age 14** | **Age 15** | **Age 16** | **Age 17** |
| --- | --- | --- | --- | --- | --- | --- | --- | --- | --- |
| Age 8 | - |  |  |  |  |  |  |  |  |
| Age 9 | 0.046 | - |  |  |  |  |  |  |  |
| Age 11 | 0.528 | 0.845 | - |  |  |  |  |  |  |
| Age 12 | 0.365 | 0.940 | 0.878 | - |  |  |  |  |  |
| Age 13 | 0.362 | 0.135 | 0.315 | 0.16 | - |  |  |  |  |
| Age 14 | 0.554 | 0.899 | 0.798 | 0.859 | 0.241 | - |  |  |  |
| Age 15 | 0.319 | 0.753 | 0.996 | 0.809 | 0.138 | 0.695 | - |  |  |
| Age 16 | 0.156 | 0.508 | 0.855 | 0.582 | 0.067 | 0.488 | 0.791 | - |  |
| Age 17 | 0.147 | 0.438 | 0.773 | 0.491 | 0.063 | 0.413 | 0.676 | 0.856 | - |
| **LPT** | **Age 8** | **Age 9** | **Age 11** | **Age 12** | **Age 13** | **Age 14** | **Age 15** | **Age 16** | **Age 17** |
| Age 8 | - |  |  |  |  |  |  |  |  |
| Age 9 | 0.728 | - |  |  |  |  |  |  |  |
| Age 11 | 0.528 | 0.745 | - |  |  |  |  |  |  |
| Age 12 | 0.590 | 0.787 | 0.989 | - |  |  |  |  |  |
| Age 13 | 0.575 | 0.769 | 0.993 | 0.982 | - |  |  |  |  |
| Age 14 | 0.337 | 0.581 | 0.915 | 0.909 | 0.933 | - |  |  |  |
| Age 15 | 0.763 | 0.938 | 0.661 | 0.706 | 0.689 | 0.478 | - |  |  |
| Age 16 | 0.928 | 0.732 | 0.551 | 0.572 | 0.557 | 0.387 | 0.738 | - |  |
| Age 17 | 0.798 | 0.932 | 0.676 | 0.711 | 0.694 | 0.503 | 0.986 | 0.763 | - |
| **MT/ISS** | **Age 8** | **Age 9** | **Age 11** | **Age 12** | **Age 13** | **Age 14** | **Age 15** | **Age 16** | **Age 17** |
| Age 8 | - |  |  |  |  |  |  |  |  |
| Age 9 | 0.840 | - |  |  |  |  |  |  |  |
| Age 11 | 0.730 | 0.923 | - |  |  |  |  |  |  |
| Age 12 | 0.871 | 0.794 | 0.720 | - |  |  |  |  |  |
| Age 13 | 0.200 | 0.252 | 0.216 | 0.346 | - |  |  |  |  |
| Age 14 | 0.611 | 0.592 | 0.53 | 0.761 | 0.545 | - |  |  |  |
| Age 15 | 0.678 | 0.645 | 0.579 | 0.826 | 0.483 | 0.932 | - |  |  |
| Age 16 | 0.552 | 0.548 | 0.485 | 0.721 | 0.549 | 0.972 | 0.900 | - |  |
| Age 17 | 0.386 | 0.429 | 0.369 | 0.601 | 0.602 | 0.870 | 0.792 | 0.894 | - |
| **MPT** | **Age 8** | **Age 9** | **Age 11** | **Age 12** | **Age 13** | **Age 14** | **Age 15** | **Age 16** | **Age 17** |
| Age 8 | - |  |  |  |  |  |  |  |  |
| Age 9 | 0.208 | - |  |  |  |  |  |  |  |
| Age 11 | 0.566 | 0.096 | - |  |  |  |  |  |  |
| Age 12 | 0.806 | 0.099 | 0.550 | - |  |  |  |  |  |
| Age 13 | 0.572 | 0.177 | 0.940 | 0.675 | - |  |  |  |  |
| Age 14 | 0.491 | 0.307 | 0.791 | 0.507 | 0.766 | - |  |  |  |
| Age 15 | 0.375 | 0.573 | 0.528 | 0.354 | 0.514 | 0.723 | - |  |  |
| Age 16 | 0.971 | 0.075 | 0.410 | 0.754 | 0.451 | 0.343 | 0.227 | - |  |
| Age 17 | 0.780 | 0.279 | 0.794 | 0.935 | 0.820 | 0.684 | 0.514 | 0.732 | - |

Note. Watson-Williams F-test

Abbreviations: LRT = lateroretrusion; LPT = lateroprotrusion; MT/ISS: mediotrusion and immediate sideshift; MPT = medioprotrusion.

**
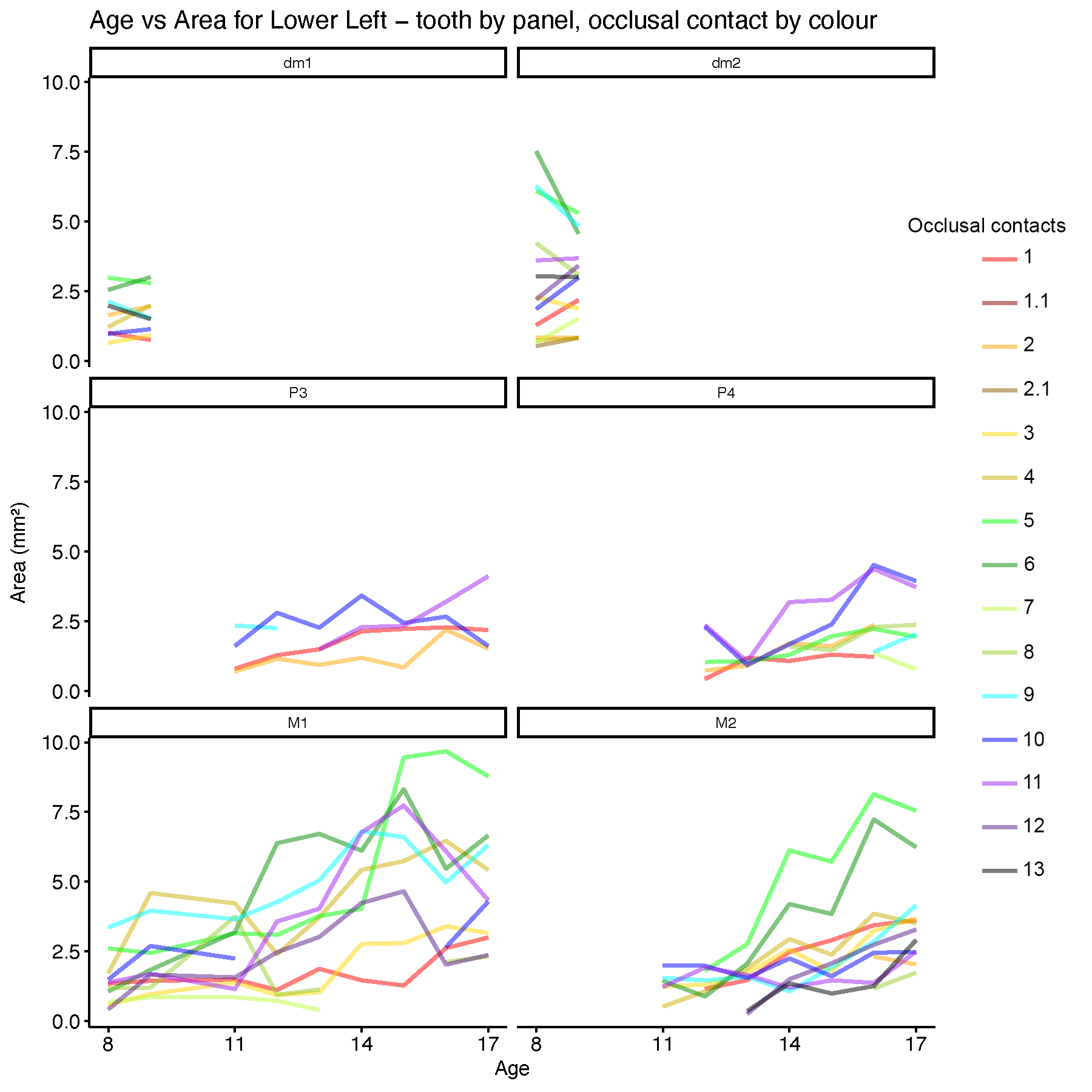
**

**S1 Fig.** Longitudinal maps of occlusal contact variation for left mandibular teeth.

**
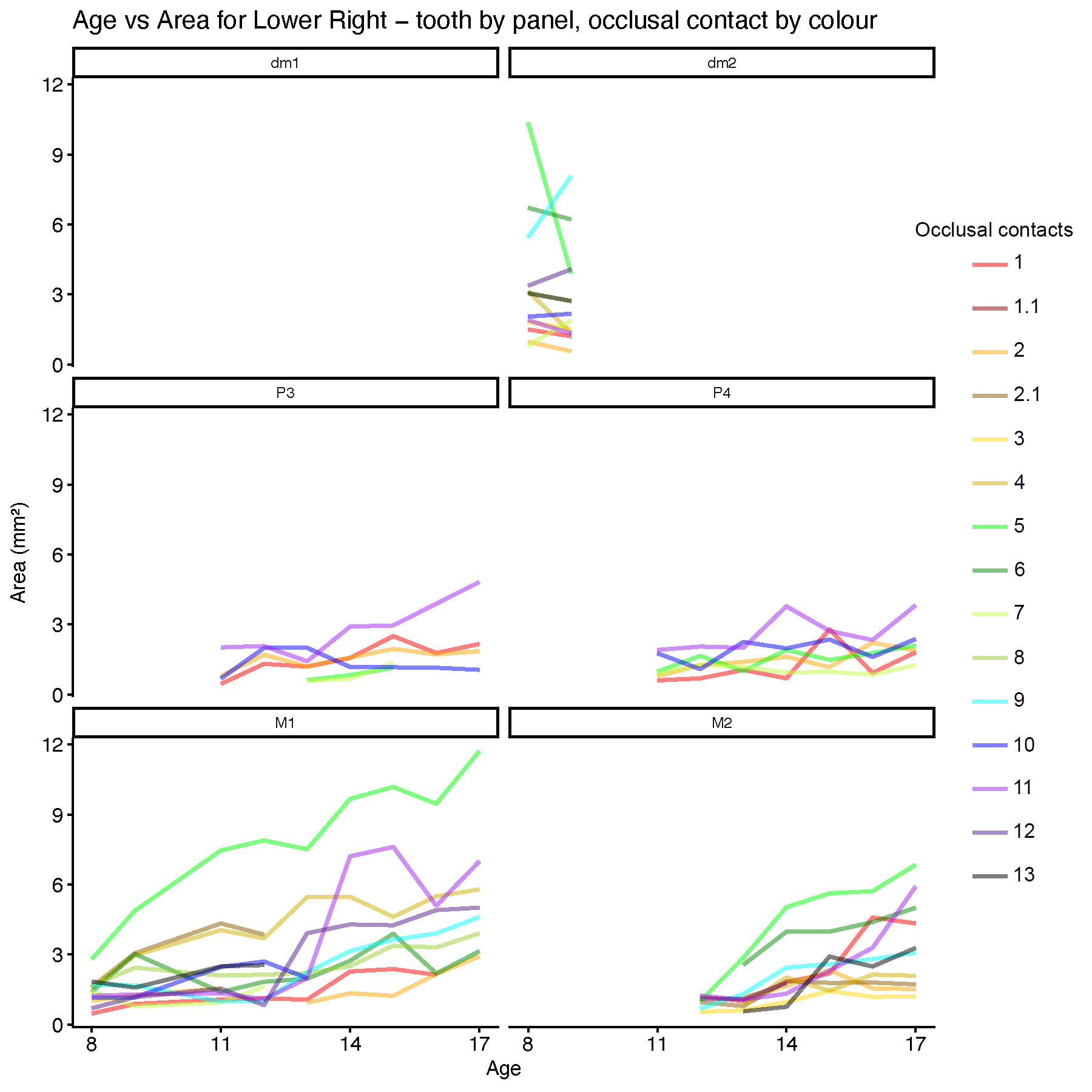
**

**S2 Fig.** Longitudinal maps of occlusal contact variation for right mandibular teeth.

**
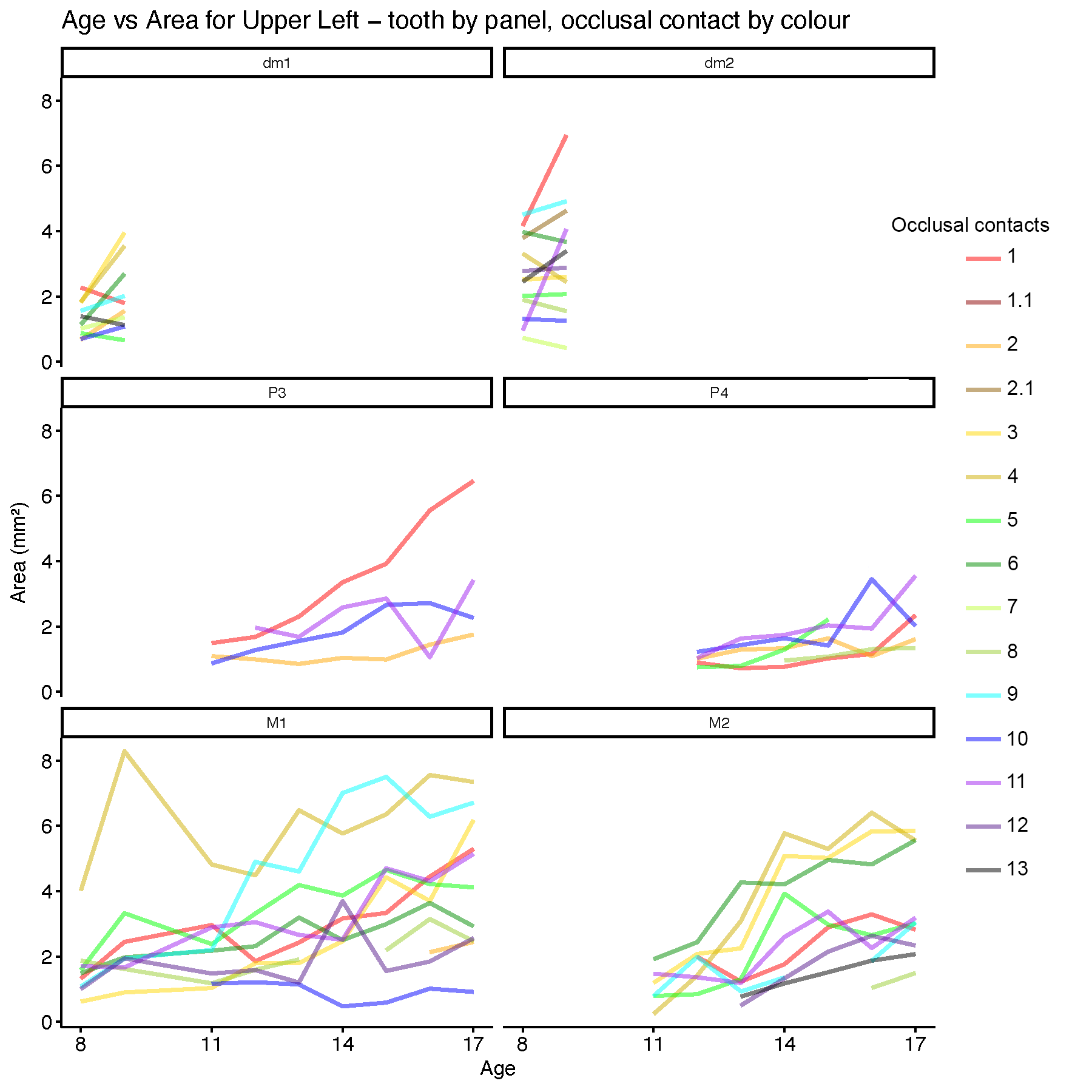
**

**S3 Fig.** Longitudinal maps of occlusal contact variation for left maxillary teeth.

**
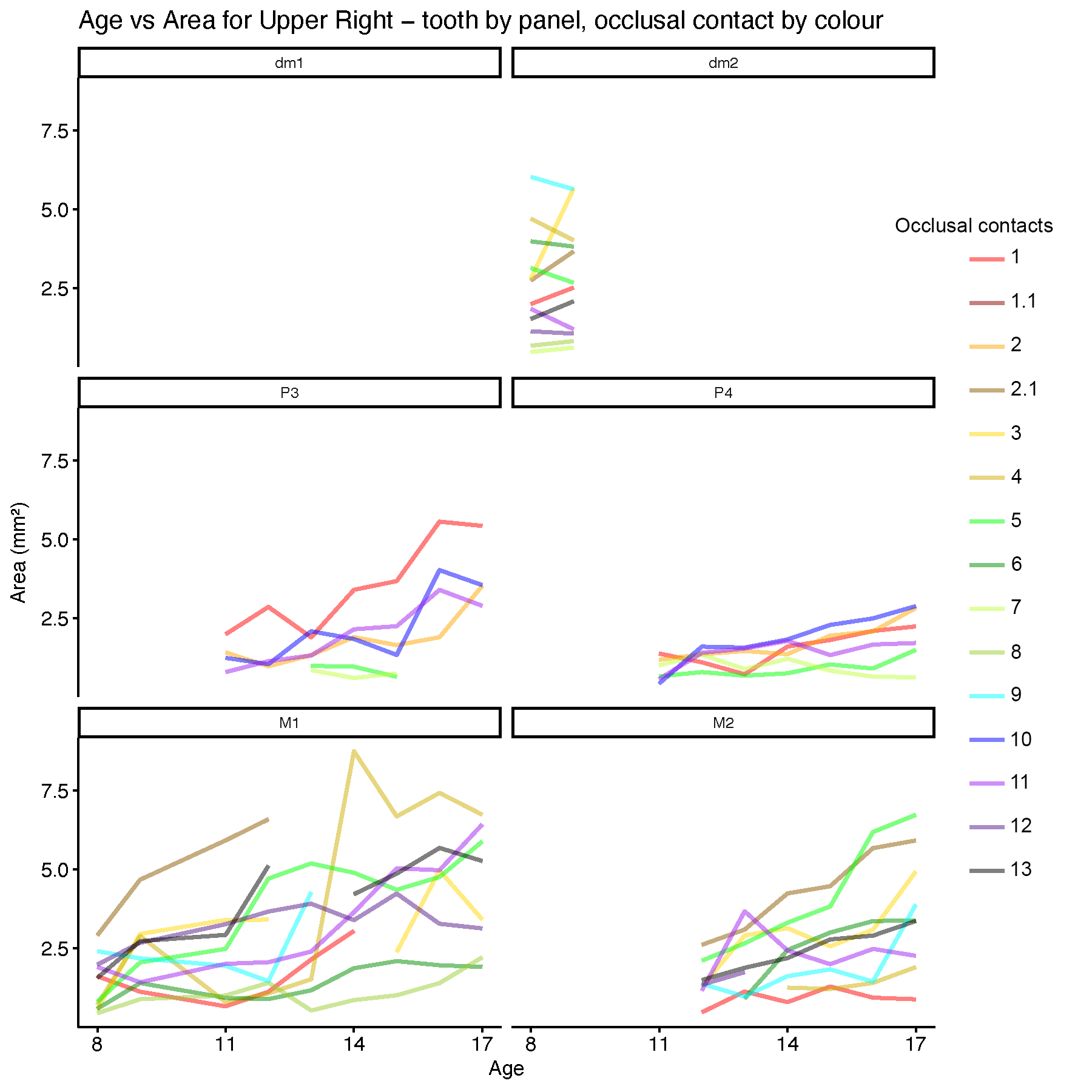
**

**S4 Fig.** Longitudinal maps of occlusal contact variation for right maxillary teeth.
